# Performance of Zero-Shot Classifiers for Categorizing RCT Abstracts by Intervention Type: Validation Study

**DOI:** 10.2196/77943

**Published:** 2026-06-18

**Authors:** Diana Buitrago-Garcia, Delphine S Courvoisier, Sami Capderou, Michele Iudici, Denis Mongin

**Affiliations:** 1 Division of Rheumatology, Geneva University Hospitals and University of Geneva, Rue Alcide-Jentzer 22, Genève, 1205, Switzerland, 41 022 372 36 78; 2 Surgery Department, Geneva University Hospitals and University of Geneva, Geneva, Geneva, Switzerland

**Keywords:** large language models, LLM, automation tools, evidence synthesis, methodology

## Abstract

**Background:**

Artificial intelligence has gained relevance due to its potential to reduce the workload in evidence synthesis or bibliometric projects. While the main focus has been lately on the use of instruction-tuned large language models, zero-shot classification models have not been tested for such task. These models are large language models trained on large datasets of labeled data able to categorize text among proposed labels, irrespective of the text domain or the topic. They are relatively small, able to run on consumer-grade computers, and almost hyperparameter-free.

**Objective:**

In our study, we use abstracts of randomized clinical trials in rheumatology as a case example to evaluate the performance of openly available, generalist, zero-shot classification models in classifying types of interventions against a human gold standard.

**Methods:**

We classified all rheumatology RCT abstracts published between 2009 and 2022 (n=1,054) as “drug” or “non-drug” using two zero-shot text classification models (DeBERTa and BART) and few-shot prompting using Llama3 8B. Different labeling of categories provided to the zero-shot classification models and different prompts provided to Llama3 8B were tested. Performance was evaluated using accuracy and predictive value of both categories against a human gold standard.

**Results:**

Most randomized controlled trials, RCTs (452/1054, 42.9%) assessed drug interventions. The DeBERTa model achieved the highest accuracy (929/1054, 88.1%; 95% CI 86%‐90%) when using the “drug” and “non-drug” labels. Llama3 8B and few-shot prompting had slightly higher accuracy and predictive values. Both zero-shot and Llama3 8B models had performance on par with a human without experience in evidence synthesis (905/1054, 85.9%; 95% CI 83.6%‐87.8% accuracy). Misclassifications occurred for trials where the intervention was harder to classify, such as procedures (eg, intra-articular injections), food compounds, vitamins, supplements, or biological treatments.

**Conclusions:**

This study shows the potential of zero-shot classification models for simple classification tasks, demonstrating accuracy comparable to that of an untrained human. These models are potential tools to streamline systematic review tasks for bibliometric studies in classifying abstracts by supplementing one reviewer.

## Introduction

The study of scientific publications is at the core of bibliometric research on evolving trends [1] and supports evidence synthesis addressing key questions in clinical and public health decision-making [2].

Abstract classification, the first step in many review processes, typically requires two independent reviewers to minimize errors and bias, with a third resolving disagreements [3,4]. This procedure is time-consuming [5] and creates a considerable burden for review teams in projects addressing broad questions.

Advances in artificial intelligence (AI) have introduced new opportunities to reduce workload, especially for abstract and title screening tasks [6]. Two main categories of AI tools have been used to date. The first includes machine learning algorithms trained directly for a specific classification task. This approach has demonstrated good and reproducible performance [7] but it lacks versatility and requires task-specific training. The second category involves prompting large language models [8,9]. While they are highly versatile and not dependent on task-specific training, their performance can vary depending on the prompts provided, sampling parameters, and their use requires substantial computing power, or a paid private Application Programming Interface (API).

Zero-shot classification models [10] represent a third, less explored option with potential advantages. These language models, constrained to assigning a probability to a label for a given text, are fine-tuned on large datasets of labeled data. They subsequently develop natural language inference capabilities allowing them to categorize text into user-defined labels without domain-specific training. Although they can be further fine-tuned, we focus here on generalist zero-shot models that are widely available and not trained on a specific domain. They are simple to use, require minimal computational resources, have no tunable input hyperparameters beyond the label set, and have broad applicability. They therefore offer both the versatility required for abstract screening and the reproducibility needed in research. Previous studies have reported encouraging results in classifying texts [10] and heterogeneous results have been reported for abstract screening in systematic reviews [11].

In our study, we use abstracts of randomized clinical trials in rheumatology as a case example to evaluate the performance of openly available, generalist, zero-shot classification models in classifying types of interventions against a human gold standard. As a benchmark, we compare its performance with the lightest version of Llama 3 available at the time of the study (8 billion parameters).

## Methods

We used an existing database of all primary reports of RCTs published in rheumatology between 2009 to 2022. Full details about search methodology can be found in a published report [12] (Multimedia Appendix 1).

### Data Extraction

One reviewer with no previous experience in evidence synthesis or rheumatology (SC) extracted information of RCTs according to the type of intervention. A second reviewer with experience in evidence synthesis (DBG) and rheumatology reviewed the data classification of the first reviewer. Disagreements were resolved by a trained rheumatologist (MI). The classification obtained was considered the gold standard.

The type of intervention, pharmacological or nonpharmacological (*drug or non-drug intervention*) was extracted for each abstract. RCTs assessing nondrug interventions [13,14] were further categorized as: Behavioral, Biological treatments, Delivery of health care services, Device, Education, Exercise therapy, Food/plants/supplements, Procedure, Surgical, Wellness and spa, and Other (Table S1 in Multimedia Appendix 1).

### Ethical Considerations

The Geneva Research Ethics Committee exempted the present study from formal ethics review since it is based on publicly available data.

### Language Models

#### Zero-Shot Classifiers

RCTs were classified by type of intervention (“*drug”* or *“non-drug*”) using the two most popular zero-shot classifiers [10] available at the HuggingFace platform [15], namely a classifier based on the DeBERTa model by Microsoft [16,17], and another classifier developed by Meta (*bart-large-mnli, based on BART* [18,19]). Both models have a total size of less than half a billion parameters.

**Figure 1.**
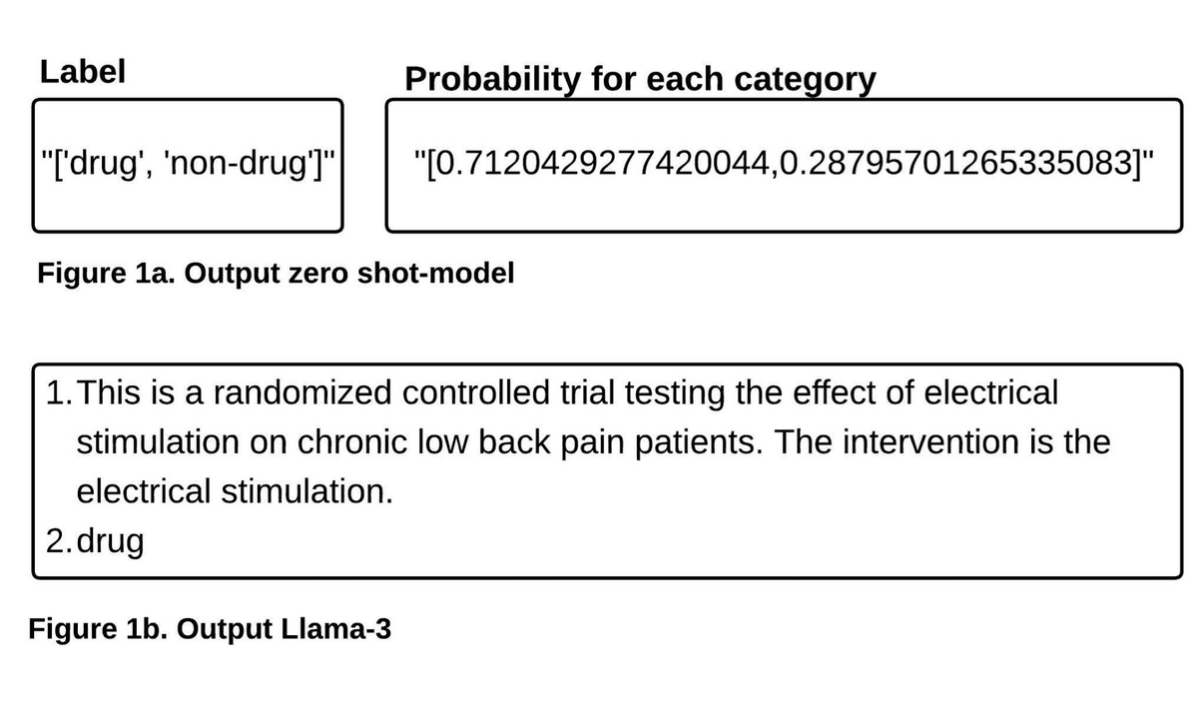
Example of the outputs of the zero-shot classifiers (**A**) and of Llama3 8B LLM (**B**) when provided a rheumatology RCT abstract. RCT: randomized controlled trial.

Zero-shot models only require labels to classify the text and provide a probability for each label (Figure 1). Abstracts were assigned to the intervention with the highest probability. To assess whether the wording of the labels could affect the classification performance, we tested seven different label combinations: the first five had two labels (drug intervention/nondrug intervention, drug intervention/other, pharmacological treatment/ nonpharmacological treatment, pharmacological treatment/ other, drug/nondrug), and the last two had the details of the “non-drug” categories (Table S2-S3 in Multimedia Appendix 1).

#### Large Language Models

We used the 8B (8 Billion parameters) version of Meta Llama 3 [20] quantized in 8bit, using a prompt with few-shot prompting with chain-of-thought method [21]. The prompt specified a structured output to extract the answer, asking to provide first an explanation after an “R:” mark, and then the classification after an “A:” mark. Output not properly formatted or providing categories not listed in the initial prompt were considered as missing and were considered as wrong in all performance metrics. The parameters used are based on a previous study [22]: a temperature of 0.3, cumulative probability and most likely next word sampling (top_p and top_k), with top_k=40 and top_p=0.95. Models were run on computer with an NVIDIA Titan X with 12 GB of VRAM, in conjunction with 20G of RAM and a standard CPU. Inference was performed using Python 3.10.4, numpy 1.22.3, pandas 1.4.2, and transformers 4.57.1. The data and prompts used are publicly available in a Gitlab repository [23].

### Data Analysis

Performance of each model and of the nontrained reviewer (SC) was assessed using four metrics:

- accuracy, defined as the sum of correct outcomes according to the gold standard divided by the total number of abstracts classified.
- Predictive value for the study being about drugs (PVD), defined as the proportion of abstracts properly classified as drug intervention.
- Predictive value for the study being about nondrugs (PVND), defined as the proportion of abstracts properly classified as nondrug intervention.
- Macro F1, defined as the average of the F1 obtained for the classification of each label (drug and nondrug). F1 is the harmonic mean of the precision and recall.

For all the metrics, the denominator includes all abstracts, even those for which the algorithm provided badly formatted outputs. Data were summarized using frequencies and percentages for categorical variables and median and interquartile range for continuous variables. Model performances were compared to those of the first reviewer.

Confidence intervals were computed using the Wilson method [24]. The analysis was done using R software (version 4.2.0; R Foundation for Statistical Computing) [25].

## Results

Of the 1054 RCTs included, 452/1054 (42.9%) assessed drug interventions and 602/1054 (57.1%) nondrug interventions. Among nondrug interventions, the most common were exercise therapy (147/602, 24.4%), procedures (144/602, 23.9%), and delivery of care (88/602, 14.3%) (Table 1).

Zero-shot classifiers took 0.3 Gb of VRAM and around 10 seconds per abstract to run, while the quantized version of Llama3 8B took 10 Gb of VRAM, and 4 seconds per abstract.

The zero-shot classification DeBERTa-based model achieved the highest accuracy using “drug” or “non-drug” categories, with an accuracy of 88.1% (95% CI 86.0%‐90.0%), predictive value for the drug category (PVD) of 80.6% (77.0‐83.7%) and a predictive value for the nondrug category (PVND) of 96.0% (95% CI 93.9%‐97.3%) and a macro F1 of 88.1% (86.7%‐89.5%) (Table 2, supplementary Tables S4 and S5 in Multimedia Appendix 1), but the accuracy varied substantially according to the label wording. The BART-based model was less affected by the wording of the labels. The highest accuracy was achieved with the “drug intervention,” “non-drug intervention” labels (prompt 1 in Table 2) with an accuracy of 86.9% (95% CI 84.7%‐88.8%), PVD 81.2% (95% CI 77.5%‐84.3%), PVND of 92.2% (95% CI 89.6%‐94.1%) and a macro F1 of 86.8% (85.3%‐88.2%) (Table 2, supplementary Tables S4 and S5 in Multimedia Appendix 1). The approaches based on Llama3 8B and few-shot prompting reached 90.8% (88.9%‐92.4%) accuracy with a prompt requiring a classification into 11 category and providing no context. Less than 2% of the output were not properly formatted and were considered as missing. The predictive values were 89.2% (86.0%‐91.7%) for the drug category and of 92.0% (89.6%‐93.9%) for the nondrug category, reaching a macro F1 of 90.6% (89.3%‐91.9%) (Table 2, supplementary Tables S4 and S5 in Multimedia Appendix 1). Adding context (prompt 1 vs 2, or prompt 3 vs 4) improved the performance of the LLM classification only for the two-category approach, where the confidence intervals of accuracy did not overlap (prompt 3: 89.3%; 87.3%‐91.0%) vs prompt 4: 85.5%; 83.2%‐87.5%). Using only two categories yielded a lower PVD but a higher PVND compared to 11-category prompts.

**Table 1.** Interventions assessed in the 1054 rheumatology RCTs^a^ included in this study.

Intervention	RCTs (N=1054), n (%)^b^
Drug interventions	452 (42.9)
Nondrug interventions	
Exercise therapy	147 (13.9)
Procedure	144 (13.7)
Delivery of health care services	88 (8.3)
Device	50 (4.7)
Food/plants/supplements	45 (4.3)
Education	31 (2.9)
Surgical	27 (2.6)
Wellness and spa	22 (2.1)
Behavioral	19 (1.8)
Biological treatments	16 (1.5)
Other	13 (1.2)

a RCT: randomized controlled trials.

b Note: Percentages may not total exactly 100 due to rounding

**Table 2.** Performance metrics of zero-shot classifiers and Llama3 8B in classifying RCT^a^ abstract between “drug” and “non-drug” intervention, for various labeling strategies (for the zero-shot classifiers) and prompting approaches (for Llama3 8B, see supplementary table S2 in Multimedia Appendix 1).

Model	Prompt/ Labeling strategy^b^	Accuracy^c^	Predictive value drugs (PVD)^d^% [95% CI]	Predictive value nondrugs (PVND)^e^% [95% CI]	Macro F1% [95% CI^f^]	Badly formatted output
Zero-shot						
DeBERTa	1	82.5% [80.1‐84.7%]	71.5% [67.8‐74.9%]	98.6% [97.0‐99.4%]	82.5% [80.9‐84.2%]	
	2	70.3% [67.5‐73%]	59.1% [55.6‐62.6%]	99.3% [97.5‐99.8%]	69.6% [67.6‐71.6%]	
	3	84.3% [81.9‐86.3%]	73.6% [69.9‐77%]	98.7% [97.1‐99.4%]	84.3% [82.7‐85.8%]	
	4	78.4% [75.8‐80.7%]	66.6% [63.0‐70.1%]	99.2% [97.7‐99.7%]	78.3% [76.5‐80.0%]	
	5	88.1% [86.0‐90.0%]	80.6% [77.0‐83.7%]	96.0% [93.9‐97.3%]	88.1% [86.7‐89.5%]	
	6	82.4% [79.9‐84.5%]	72.4% [68.7‐75.8%]	95.2% [92.9‐96.8%]	82.4% [80.7‐84.0%]	
	7	69.6% [66.8‐72.3%]	81.1% [75.3‐85.8%]	66.7% [63.5‐69.8%]	64.8% [62.6‐67.0%]	
BART	1	86.9% [84.7‐88.8%]	81.2% [77.5‐84.3%]	92.2% [89.6‐94.1%]	86.8% [85.3‐88.2%]	
	2	85.4% [83.1‐87.4%]	76.3% [72.7‐79.6%]	95.9% [93.8‐97.3%]	85.4% [83.9‐86.9%]	
	3	86.3% [84.1‐88.3%]	82.6% [78.9‐85.8%]	89.3% [86.6‐91.6%]	86.1% [84.6‐87.6%]	
	4	86.1% [83.9‐88.1%]	78.5% [74.9‐81.8%]	94% [91.6‐95.8%]	86.1% [84.6‐87.6%]	
	5	84.9% [82.6‐86.9%]	82.9% [79.1‐86.1%]	86.4% [83.4‐88.9%]	84.6% [83.0‐86.1%]	
	6	86.1% [83.9‐88.1%]	79.8% [76.1‐83.0%]	92.2% [89.7‐94.2%]	86.1% [84.6‐87.5%]	
	7	77.8% [75.2‐80.2%]	83.6% [79.2‐87.3%]	75.2% [71.9‐78.2%]	76.1% [74.2‐78.0%]	
Llama3 8B						
	1	89.5% [87.5‐91.2%]	88% [84.6‐90.7%]	90.6% [88.0‐92.7%]	89.2% [87.9‐90.6%]	3.1% [2.2‐4.4%]
	2	90.8% [88.9‐92.4%]	89.2% [86.0‐91.7%]	92.0% [89.6‐93.9%]	90.6% [89.3‐91.9%]	1.9% [1.2‐2.9%]
	3	89.3% [87.3‐91.0%]	82.2% [78.7‐85.2%]	96.4% [94.4‐97.7%]	89.2% [87.9‐90.6%]	0.3% [0.1‐0.8%]
	4	85.5% [83.2‐87.5%]	79% [75.3‐82.3%]	91.7% [89.0‐93.7%]	85.4% [83.9‐86.9%]	5.1% [3.9‐6.6%]

a RCT: randomized controlled trial.

b Detailed information about the labels used are in supplementary Table S2, and in supplementary table S3 in Multimedia Appendix 1 for prompts.

c Sum of correct outcomes according to the gold standard divided by the total number of abstracts classified.

d Proportion of abstracts properly classified as drug intervention.

e Proportion of abstracts properly classified as nondrug intervention.

f Average of F1 for each label. F1 is the harmonic mean of precision and recall.

**Figure 2.**
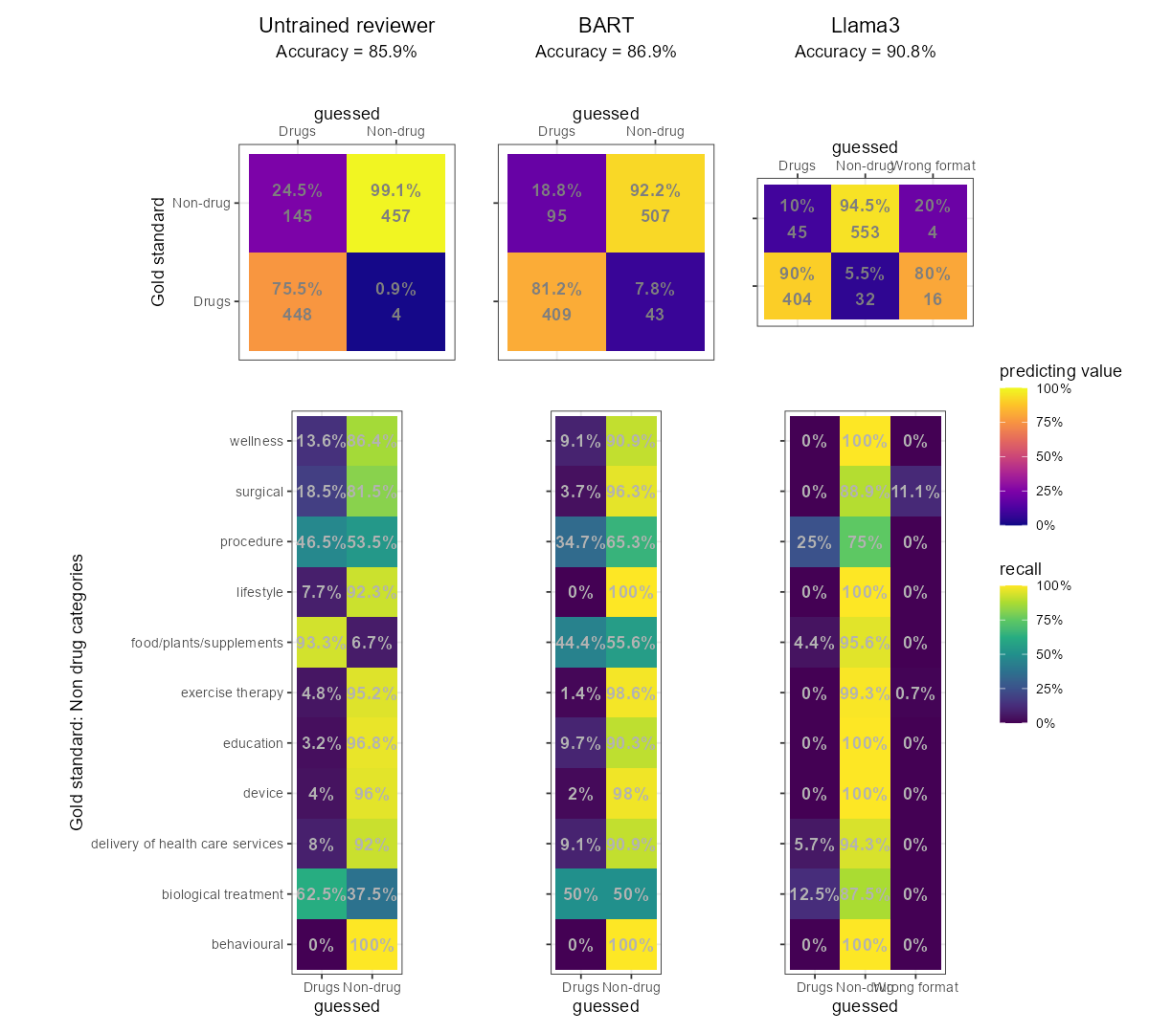
Performance metrics when classifying abstracts between “drug” or “non-drug” interventions, for the untrained reviewer (left column), the BART-based zero-shot classifier (middle column) and Llama3 8B model (right column).

The nontrained reviewer reached 85.9% accuracy (95% CI: 83.6%‐87.8%), with a PVD lower than those obtained with AI models (75.6%; 95% CI: 72.0%‐78.9%) but a higher PVND (99.1%; 95% CI 97.8%‐99.7%). When looking specifically at nondrug interventions, both the nontrained reviewer and BART had suboptimal performance for trials involving procedures (eg, intra-articular injections), food compounds, vitamins, supplements, or biological treatments. These misclassifications were not resolved by using explicit labeling of all nondrug categories (labeling strategies 6 and 7, see supplementary table S2 in Multimedia Appendix 1). Llama3 8B, aided by detailed prompts, properly classified food/supplements as nondrug and performed better in general at classifying drug interventions (Figure 2, supplementary table S6 in Multimedia Appendix 1).

When testing the zero-shot classifiers on the eleven categories of nondrug intervention, the classifier performed globally poorly with discrepant results between categories, with some, such as “surgical,” reaching as high as 92.6% (95% CI 76.6%‐97.9%) accuracy, when others such as “wellness” or “device” had a zero or close to zero accuracy (see Table S7 in Multimedia Appendix 1 for full details).

## Discussion

### Principal Findings

Our study found that zero-shot classifier models can reach 85%‐90% accuracy on a simple binary classification task and 85% macro F1, despite not being trained on medical or rheumatology-specific data. Meta’s BART model was the least sensitive to changes in label phrasing and produced the best overall performance when using simple two-category labels. Its accuracy was comparable to that of an untrained human reviewer and slightly lower than that of Llama 3 8B. Given its ease of use and small computational footprint, BART is a practical option for abstract classification tasks in resource-limited settings.

Both zero-shot classification models struggled to classify RCTs testing procedures, food/supplements, or biological treatment as drugs. These interventions resemble drug-based treatments (eg, supplements, biologics) or involve drugs administered through procedures, yet differ by requiring specialized expert actions (eg, injections, ultrasound guidance). This nuance created difficulties for both the zero-shot models and the untrained reviewer. Adjusting label phrasing did not resolve these errors, likely because zero-shot models cannot use detailed task instructions. For Llama3 8B, the effect of prompt design was more nuanced: providing context improved accuracy only in the two-category setting, while the number of categories influenced the balance between predictive values for drugs and nondrugs. It is expected that more advanced LLMs may reduce such misclassifications when provided with explicit explanatory prompts. Zero-shot classifiers were unable to reliably handle multi-label or more complex classification tasks, for which high-performance LLMs remain more suitable.

### Comparison to Prior Work

Our results align with studies evaluating RCT abstracts using trained machine learning models [26], or prompt-based LLMs [27], which suggest these methods could supplement a reviewer for simple classification tasks. Recent work [28] shows that bigger models (Llama3 70B) can reach 95% accuracy on the same classification task, albeit at substantially greater computational cost, and that aggregating outputs from multiple LLMs can exceed human-level performance. Nevertheless, expert reviewers remain essential to supervise the process and prevent inappropriate exclusion or misclassification of studies [29].

### Strengths and Limitations

This study analyzed over 1000 rheumatology RCT abstracts, enabling precise estimates of model performance. It also examined the details of the subtypes of nondrug interventions, to allow a good understanding of where humans and LLM models misclassify. The zero-shot models presented are free to use, available and stable for more than three years, running on standard computers. Besides, we provide the prompts and data used in our study, enabling replication of our methods in different medical fields.

Several limitations should be noted. First, we evaluated only a single, simple binary classification task within rheumatology. Thus, the present results may not generalize to other fields or to multi-label tasks. Second, LLMs are prone to a rapid development and better performance could be obtained using more recent models. Finally, the reference human performance is based on a single untrained reviewer, which may not reflect broader human variability, though the observed error rate aligns with typical rates reported for systematic review screening [30].

### Conclusion

Zero-shot classification models can classify with accuracy similar to a scientist untrained in evidence synthesis, perform well on simple classification tasks, while not requiring specific training for the scientific domain or high-performance computing. These tools have the potential to facilitate classification processes of systematic reviews or bibliometric studies by replacing one reviewer for simple classification tasks.

## Supplementary material

10.2196/77943 Multimedia Appendix 1 Supplementary materials.

## Abbreviations

AI: artificial intelligence
API: application programming interface
LLM: large language model
PVD: predictive value for the study being about drugs
PVND: predictive value for the study being about nondrugs
RCT: randomized controlled trial
