# Supplementary material for: Performance of Zero-Shot Classifiers for Categorizing RCT Abstracts by Intervention Type: Validation Study
Source: JMIR Med Inform. 2026 Jun 18;14:e77943. doi: 10.2196/77943 (PMC13278612; doi:10.2196/77943)
Supplement: Multimedia Appendix 1 [file medinform-v14-e77943-s001.docx]

# Supplementary material

# Zero-Shot classifiers for Classifying RCT Abstracts by Intervention Type

Authors: Diana Buitrago-Garcia^1^, Delphine S. Courvoisier^1^, Michele Iudici,^1^ Denis Mongin^1^

^1^ Division of Rheumatology, Geneva University Hospitals and University of Geneva, Geneva, Switzerland.

Appendix 1. Search strategy to identify primary reports of RCTs in rheumatology

*Search strategy for rheumatology journals*

"(0303-464X[IS] OR 2523-3106[IS] OR 0341-051X[IS] OR 0003-4967[IS] OR 2148-5046[IS] OR 1471-2474[IS] OR 0392-856X[IS] OR 0770-3198[IS] OR 1040-8711[IS] OR 1523-3774[IS] OR 1573-3971[IS] OR 1110-1164[IS] OR 2147-9720[IS] OR 0973-3698[IS] OR 1756-1841[IS] OR 1687-9260[IS] OR 1076-1608[IS] OR 1297-319X[IS] OR 2093-940X[IS] OR 0315-162X[IS] OR 2397-1983[IS] OR 2665-9913[IS] OR 0961-2033[IS] OR 2053-8790[IS] OR 1439-7595[IS] OR 1478-2189[IS] OR 1759-4790[IS] OR 1179-156X[IS] OR 1063-4584[IS] OR 1546-0096[IS] OR 2151-464X[IS] OR 1532-1770[IS] OR 0048-7449[IS] OR 1699-258X[IS] OR 1606-5581[IS] OR 0889-857X[IS] OR 2326-5191[IS] OR 1462-0324[IS] OR 2514-1775[IS] OR 2198-6576[IS] OR 0172-8172[IS] OR 2056-5933[IS] OR 0300-9742[IS] OR 0049-0172[IS] OR 1759-720X[IS] OR 1478-6354[IS] OR 2147-2653[IS] OR 0340-1855[IS] OR 0004-3591[IS]) AND 2009:2022 [DP] AND (trial[TI] OR rct[TI]) AND (randomized[TI] OR randomised[TI]) NOT (non-randomized[TI] OR non-randomised[TI] OR nonrandomized[TI] OR nonrandomised[TI]) NOT protocol[TI] NOT secondary analysis[TI] NOT (systematic-review[TI] OR meta-analysis[TI]) NOT (design[TI] AND methodology[TI])"

*Search strategy for general medicine journals*

"(1474-547X[IS] OR 1533-4406[IS] OR 1538-3598[IS] OR 1756-1833[IS] OR 1539-3704[IS]) AND 2009:2022 [DP] AND (trial[TI] OR rct[TI]) AND (randomized[TI] OR randomised[TI]) NOT (non-randomized[TI] OR non-randomised[TI] OR nonrandomized[TI] OR nonrandomised[TI]) NOT protocol[TI] NOT secondary analysis[TI] NOT (systematic-review[TI] OR meta-analysis[TI]) NOT (design[TI] AND methodology[TI]) AND (Vasculitis[Mesh] OR Polychondritis, Relapsing[Mesh] OR Dermatomyositis[Mesh] OR Lupus Erythematosus, Systemic[Mesh] OR Mixed Connective Tissue Disease[Mesh] OR Rheumatic Diseases[Mesh] OR Polymyositis[Mesh] OR Arthritis, Rheumatoid[Mesh] OR Spondylarthritis[Mesh] OR Osteoarthritis[Mesh] OR Crystal Arthropathies[Mesh] OR gout[tiab] OR chondrocalc*[tiab] OR Wegener[tiab] OR churg-strauss[tiab] OR granulomatosis[tiab] OR polyangiitis[tiab] OR *angiitis[tiab] OR polyarteritis[tiab] OR Takayasu[tiab] OR Horton[tiab] OR aortitis[tiab] OR giant cell[tiab] OR polymyalgia[tiab] OR scleroderma[tiab] OR systemic sclerosis[tiab] OR lupus[tiab] OR sjogren[tiab] OR dermatomyositis[tiab] OR polymyositis[tiab] OR undifferentiated connective tissue[tiab] OR UCTD[tiab] OR mixed connective tissue[tiab] OR MCTD[tiab] OR behcet[tiab] OR cogan[tiab] OR Schoenlein-Henoch[tiab] OR rheumatoid[tiab] OR Still disease[tiab] OR osteoarthr*[tiab] OR spondyl*[tiab] OR reactive arthritis[tiab] OR psoriatic arthritis[tiab] OR arthrose[tiab] OR arthritis[tiab])"

Table S1. Definitions used to classify RCTs addressing non-pharmacological interventions

| **Sub-Group** | **Definition** |
| --- | --- |
| Behavioural | Interventions designed to affect the actions that individuals take regarding their health. It involves using behaviour analytic techniques to change, reduce or modify conducts in people. |
| Biological treatments | Interventions assessing the effect of blood components or cell therapies. |
| Delivery of health care services | Delivery of services within any a health system where patients receive the treatment and supplies. |
| Device | Interventions involving any instrument, apparatus, implement, machine, appliance, implant, reagent for in vitro use, software, material or other similar or related article, intended by the manufacturer to be used, alone or in combination for a medical purpose. |
| Education | Interventions that involved providing teaching, instruction or pedagogy activities to patients or other populations. |
| Exercise therapy | Interventions that assess the effect of sports or any physical training. |
| Food/plants/supplements | Any food, herbal or dietary supplements that are not classified as a drug. |
| Procedure | Interventions that involve any practice of a health practitioner that assesses a combination of special skills or abilities and may require drugs, devices, or both. |
| Surgical | Invasive interventions that require an incision, cutting into the skin, to access body tissue, organs, or other internal parts. |
| Wellness and spa | Interventions that assess the effectiveness of thermal baths, spas or massage therapies. |
| Other | Other interventions that do not fit into the former categories |

Table S2. Detail of the labelling tested for the zero-shot models

| **Labelling strategy** | **Category tested** |
| --- | --- |
| 1 | "drug intervention","non-drug intervention" |
| 2 | "drug intervention","other" |
| 3 | "pharmacological treatment","non-pharmacological treatment" |
| 4 | "pharmacological treatment","other" |
| 5 | "drug","non-drug" |
| 6 | "drug intervention", “Behavioural intervention”, “Biological treatments”, “Delivery of health care services change”, “Device test”, “Education intervention”, “Exercise therapy intervention”, “Food/plants/supplements treatments”, “Procedure”, “Surgical intervention”, “Wellness and spa intervention”, and “Other” |
| 7 | "drug","behavioral","health Care delivery","wellness","device","educational","physiotherapeutic","food and plant","lifestyle","surgical","procedure","biological treatments" |

**Table S3: Structure of the prompts tested for Llama3 8B**

| **Prompt** | **Description** |
| --- | --- |
| 1 | context, 11 categories (“Behavioural intervention”, “Biological treatments”, “Delivery of health care services change”, “Device test”, “Education intervention”, “Exercise therapy intervention”, “Food/plants/supplements treatments”, “Procedure”, “Surgical intervention”, “Wellness and spa intervention”, and “Other”) |
| 2 | no context, 11 categories  (“Behavioural intervention”, “Biological treatments”, “Delivery of health care services change”, “Device test”, “Education intervention”, “Exercise therapy intervention”, “Food/plants/supplements treatments”, “Procedure”, “Surgical intervention”, “Wellness and spa intervention”, and “Other”) |
| 3 | context 2 categories (“Drug” “non-drug”) |
| 4 | no context 2 categories (“Drug” “non-drug”) |

**Table S4. Performance metrics when predicting the drug category, for the different models tested and labelling/prompting strategies**

| **Model** | **Labelling strategy/ prompt** | **TP** | **FN** | **TN** | **FP** | **Precision** | **Recall** | **Accuracy** | **F1-Scores** |
| --- | --- | --- | --- | --- | --- | --- | --- | --- | --- |
| DeBERTa | 1 | 446 | 6 | 424 | 178 | 71.5% [67.8-74.9%] | 98.7% [97.1-99.4%] | 82.5% [80.1-84.7%] | 82.9% [80.5-85%] |
|  | 2 | 450 | 2 | 291 | 311 | 59.1% [55.6-62.6%] | 99.6% [98.4-99.9%] | 70.3% [67.5-73%] | 74.2% [71.7-76.6%] |
|  | 3 | 446 | 6 | 442 | 160 | 73.6% [69.9-77%] | 98.7% [97.1-99.4%] | 84.3% [81.9-86.3%] | 84.3% [82-86.4%] |
|  | 4 | 449 | 3 | 377 | 225 | 66.6% [63-70.1%] | 99.3% [98.1-99.8%] | 78.4% [75.8-80.7%] | 79.8% [77.3-82%] |
|  | 5 | 431 | 21 | 498 | 104 | 80.6% [77-83.7%] | 95.4% [93-96.9%] | 88.1% [86-90%] | 87.3% [85.1-89.3%] |
|  | 6 | 430 | 22 | 438 | 164 | 72.4% [68.7-75.8%] | 95.1% [92.7-96.8%] | 82.4% [79.9-84.5%] | 82.2% [79.8-84.4%] |
|  | 7 | 172 | 280 | 562 | 40 | 81.1% [75.3-85.8%] | 38.1% [33.7-42.6%] | 69.6% [66.8-72.3%] | 51.8% [48-55.6%] |
| BART | 1 | 409 | 43 | 507 | 95 | 81.2% [77.5-84.3%] | 90.5% [87.4-92.9%] | 86.9% [84.7-88.8%] | 85.6% [83.2-87.7%] |
|  | 2 | 432 | 20 | 468 | 134 | 76.3% [72.7-79.6%] | 95.6% [93.3-97.1%] | 85.4% [83.1-87.4%] | 84.9% [82.5-86.9%] |
|  | 3 | 390 | 62 | 520 | 82 | 82.6% [78.9-85.8%] | 86.3% [82.8-89.2%] | 86.3% [84.1-88.3%] | 84.4% [81.9-86.6%] |
|  | 4 | 421 | 31 | 487 | 115 | 78.5% [74.9-81.8%] | 93.1% [90.4-95.1%] | 86.1% [83.9-88.1%] | 85.2% [82.9-87.3%] |
|  | 5 | 369 | 83 | 526 | 76 | 82.9% [79.1-86.1%] | 81.6% [77.8-84.9%] | 84.9% [82.6-86.9%] | 82.3% [79.6-84.6%] |
|  | 6 | 410 | 42 | 498 | 104 | 79.8% [76.1-83%] | 90.7% [87.7-93.1%] | 86.1% [83.9-88.1%] | 84.9% [82.5-87%] |
|  | 7 | 271 | 181 | 549 | 53 | 83.6% [79.2-87.3%] | 60% [55.4-64.4%] | 77.8% [75.2-80.2%] | 69.8% [66.5-73%] |
| Llama3 8B | 1 | 395 | 57 | 548 | 54 | 88% [84.6-90.7%] | 87.4% [84-90.1%] | 89.5% [87.5-91.2%] | 87.7% [85.4-89.7%] |
|  | 2 | 404 | 48 | 553 | 49 | 89.2% [86-91.7%] | 89.4% [86.2-91.9%] | 90.8% [88.9-92.4%] | 89.3% [87.1-91.1%] |
|  | 3 | 433 | 19 | 508 | 94 | 82.2% [78.7-85.2%] | 95.8% [93.5-97.3%] | 89.3% [87.3-91%] | 88.5% [86.3-90.3%] |
|  | 4 | 407 | 45 | 494 | 108 | 79% [75.3-82.3%] | 90% [86.9-92.5%] | 85.5% [83.2-87.5%] | 84.2% [81.7-86.3%] |

**Table S5. Performance metrics when predicting the non-drug category, for the different models tested and labelling/prompting strategies**

| **Model** | **Labelling strategy/ prompt** | **TP** | **FN** | **TN** | **FP** | **Precision** | **Recall** | **Accuracy** | **F1-Scores** |
| --- | --- | --- | --- | --- | --- | --- | --- | --- | --- |
| DeBERTa | 1 | 424 | 178 | 446 | 6 | 98.6% [97-99.4%] | 70.4% [66.7-73.9%] | 82.5% [80.1-84.7%] | 82.2% [79.7-84.4%] |
|  | 2 | 291 | 311 | 450 | 2 | 99.3% [97.5-99.8%] | 48.3% [44.4-52.3%] | 70.3% [67.5-73%] | 65% [61.8-68.1%] |
|  | 3 | 442 | 160 | 446 | 6 | 98.7% [97.1-99.4%] | 73.4% [69.8-76.8%] | 84.3% [81.9-86.3%] | 84.2% [81.9-86.3%] |
|  | 4 | 377 | 225 | 449 | 3 | 99.2% [97.7-99.7%] | 62.6% [58.7-66.4%] | 78.4% [75.8-80.7%] | 76.8% [74-79.3%] |
|  | 5 | 498 | 104 | 431 | 21 | 96% [93.9-97.3%] | 82.7% [79.5-85.5%] | 88.1% [86-90%] | 88.8% [86.9-90.6%] |
|  | 6 | 438 | 164 | 430 | 22 | 95.2% [92.9-96.8%] | 72.8% [69.1-76.2%] | 82.4% [79.9-84.5%] | 82.5% [80.1-84.7%] |
|  | 7 | 562 | 40 | 172 | 280 | 66.7% [63.5-69.8%] | 93.4% [91.1-95.1%] | 69.6% [66.8-72.3%] | 77.8% [75.6-79.9%] |
| BART | 1 | 507 | 95 | 409 | 43 | 92.2% [89.6-94.1%] | 84.2% [81.1-86.9%] | 86.9% [84.7-88.8%] | 88% [86-89.8%] |
|  | 2 | 468 | 134 | 432 | 20 | 95.9% [93.8-97.3%] | 77.7% [74.2-80.9%] | 85.4% [83.1-87.4%] | 85.9% [83.7-87.8%] |
|  | 3 | 520 | 82 | 390 | 62 | 89.3% [86.6-91.6%] | 86.4% [83.4-88.9%] | 86.3% [84.1-88.3%] | 87.8% [85.9-89.6%] |
|  | 4 | 487 | 115 | 421 | 31 | 94% [91.6-95.8%] | 80.9% [77.6-83.8%] | 86.1% [83.9-88.1%] | 87% [84.9-88.8%] |
|  | 5 | 526 | 76 | 369 | 83 | 86.4% [83.4-88.9%] | 87.4% [84.5-89.8%] | 84.9% [82.6-86.9%] | 86.9% [84.9-88.7%] |
|  | 6 | 498 | 104 | 410 | 42 | 92.2% [89.7-94.2%] | 82.7% [79.5-85.5%] | 86.1% [83.9-88.1%] | 87.2% [85.2-89%] |
|  | 7 | 549 | 53 | 271 | 181 | 75.2% [71.9-78.2%] | 91.2% [88.7-93.2%] | 77.8% [75.2-80.2%] | 82.4% [80.3-84.4%] |
| Llama3 8B | 1 | 548 | 54 | 395 | 57 | 90.6% [88-92.7%] | 91% [88.5-93.1%] | 89.5% [87.5-91.2%] | 90.8% [89-92.3%] |
|  | 2 | 553 | 49 | 404 | 48 | 92% [89.6-93.9%] | 91.9% [89.4-93.8%] | 90.8% [88.9-92.4%] | 91.9% [90.3-93.3%] |
|  | 3 | 508 | 94 | 433 | 19 | 96.4% [94.4-97.7%] | 84.4% [81.3-87.1%] | 89.3% [87.3-91%] | 90% [88.1-91.6%] |
|  | 4 | 494 | 108 | 407 | 45 | 91.7% [89-93.7%] | 82.1% [78.8-84.9%] | 85.5% [83.2-87.5%] | 86.6% [84.5-88.4%] |

Table S6. Confusion matrix when predicting the interventions (drug non drug) detailed by the non-drug categories

| **Model** | **Predicted**  **True category** | **Drugs** | **Non-drug** | **Badly formatted outputs** |
| --- | --- | --- | --- | --- |
| DeBERTa  Labelling strategy 5 | Behavioural | 0 | 19 |  |
|  | Biological Treatment | 8 | 8 |  |
|  | Delivery of Health Care Services | 8 | 80 |  |
|  | Device | 1 | 49 |  |
|  | Drugs | 431 | 21 |  |
|  | Education | 1 | 30 |  |
|  | Exercise Therapy | 2 | 145 |  |
|  | Food/Plants/Supplements | 22 | 23 |  |
|  | Lifestyle | 0 | 13 |  |
|  | Procedure | 58 | 86 |  |
|  | Surgical | 2 | 25 |  |
|  | Wellness | 2 | 20 |  |
| BART Labelling strategy 1 | Behavioural | 0 | 19 |  |
|  | Biological Treatment | 8 | 8 |  |
|  | Delivery of Health Care Services | 8 | 80 |  |
|  | Device | 1 | 49 |  |
|  | Drugs | 409 | 43 |  |
|  | Education | 3 | 28 |  |
|  | Exercise Therapy | 2 | 145 |  |
|  | Food/Plants/Supplements | 20 | 25 |  |
|  | Lifestyle | 0 | 13 |  |
|  | Procedure | 50 | 94 |  |
|  | Surgical | 1 | 26 |  |
|  | Wellness | 2 | 20 |  |
| Llama 3 8B prompt 2 | Behavioural | 0 | 19 | 0 |
|  | Biological Treatment | 2 | 14 | 0 |
|  | Delivery of Health Care Services | 5 | 83 | 0 |
|  | Device | 0 | 50 | 0 |
|  | Drugs | 404 | 32 | 16 |
|  | Education | 0 | 31 | 0 |
|  | Exercise Therapy | 0 | 146 | 1 |
|  | Food/Plants/Supplements | 2 | 43 | 0 |
|  | Lifestyle | 0 | 13 | 0 |
|  | Procedure | 36 | 108 | 0 |
|  | Surgical | 0 | 24 | 3 |
|  | Wellness | 0 | 22 | 0 |

Table S7. Confusion matrix of the non-drug interventions when the labelling strategy include all non-drug categories

| **Type of study** | **True label**  **Predicted** | **Behavioural** | **Biological Treatment** | **Delivery of Health Care Services** | **Device** | **Drugs** | **Education** | **Exercise Therapy** | **Food/ Plants/ Supplements** | **Lifestyle** | **Procedure** | **Surgical** | **Wellness** |
| --- | --- | --- | --- | --- | --- | --- | --- | --- | --- | --- | --- | --- | --- |
| DeBERTa Labelling strategy 6 | Behavioural | 12 | 0 | 5 | 0 | 0 | 4 | 3 | 0 | 1 | 0 | 0 | 1 |
|  | Biological Treatment | 0 | 1 | 0 | 0 | 13 | 0 | 1 | 1 | 0 | 1 | 0 | 0 |
|  | Delivery of Health Care Services | 0 | 0 | 1 | 0 | 0 | 0 | 0 | 0 | 0 | 0 | 0 | 0 |
|  | Device | 0 | 0 | 0 | 4 | 0 | 0 | 0 | 0 | 0 | 0 | 0 | 0 |
|  | Drugs | 0 | 13 | 13 | 6 | 430 | 4 | 4 | 40 | 1 | 80 | 0 | 3 |
|  | Education | 4 | 0 | 6 | 1 | 0 | 16 | 11 | 0 | 0 | 0 | 0 | 0 |
|  | Exercise Therapy | 2 | 0 | 14 | 18 | 2 | 1 | 81 | 2 | 0 | 29 | 2 | 13 |
|  | Food/Plants/Supplements | 0 | 0 | 0 | 0 | 0 | 0 | 0 | 2 | 0 | 0 | 0 | 0 |
|  | Lifestyle | 0 | 0 | 0 | 1 | 0 | 0 | 0 | 0 | 7 | 0 | 0 | 0 |
|  | Procedure | 1 | 0 | 34 | 15 | 1 | 5 | 42 | 0 | 4 | 17 | 0 | 4 |
|  | Surgical | 0 | 2 | 15 | 5 | 6 | 1 | 5 | 0 | 0 | 17 | 25 | 1 |
|  | Wellness | 0 | 0 | 0 | 0 | 0 | 0 | 0 | 0 | 0 | 0 | 0 | 0 |
| DeBERTa Labelling strategy 7 | Behavioural | 3 | 0 | 1 | 0 | 0 | 2 | 0 | 0 | 0 | 0 | 0 | 0 |
|  | Biological Treatment | 0 | 11 | 5 | 0 | 226 | 2 | 0 | 14 | 0 | 19 | 0 | 0 |
|  | Delivery of Health Care Services | 3 | 0 | 31 | 4 | 5 | 6 | 15 | 0 | 7 | 3 | 0 | 3 |
|  | Device | 0 | 0 | 0 | 0 | 0 | 0 | 0 | 0 | 0 | 0 | 0 | 0 |
|  | Drugs | 0 | 2 | 2 | 0 | 172 | 0 | 0 | 17 | 0 | 18 | 0 | 1 |
|  | Education | 3 | 0 | 1 | 0 | 0 | 9 | 2 | 0 | 0 | 0 | 0 | 0 |
|  | Exercise Therapy | 4 | 1 | 14 | 28 | 29 | 5 | 101 | 9 | 2 | 65 | 3 | 14 |
|  | Food/Plants/Supplements | 0 | 0 | 0 | 0 | 0 | 0 | 0 | 2 | 0 | 0 | 0 | 0 |
|  | Lifestyle | 0 | 0 | 0 | 0 | 0 | 0 | 0 | 0 | 0 | 0 | 0 | 0 |
|  | Procedure | 6 | 2 | 22 | 13 | 7 | 5 | 28 | 3 | 4 | 20 | 0 | 3 |
|  | Surgical | 0 | 0 | 12 | 5 | 13 | 2 | 1 | 0 | 0 | 19 | 24 | 1 |
|  | Wellness | 0 | 0 | 0 | 0 | 0 | 0 | 0 | 0 | 0 | 0 | 0 | 0 |
| BART Labelling strategy 6 | Behavioural | 14 | 0 | 11 | 2 | 8 | 2 | 6 | 1 | 4 | 4 | 1 | 1 |
|  | Biological Treatment | 0 | 3 | 1 | 0 | 14 | 0 | 0 | 10 | 0 | 2 | 0 | 0 |
|  | Delivery of Health Care Services | 0 | 0 | 16 | 0 | 0 | 2 | 0 | 0 | 2 | 0 | 0 | 0 |
|  | Device | 0 | 0 | 8 | 0 | 0 | 0 | 0 | 3 | 1 | 4 | 0 | 0 |
|  | Drugs | 0 | 13 | 5 | 1 | 410 | 1 | 0 | 23 | 0 | 60 | 1 | 0 |
|  | Education | 3 | 0 | 3 | 1 | 1 | 12 | 1 | 0 | 0 | 1 | 0 | 0 |
|  | Exercise Therapy | 1 | 0 | 13 | 32 | 4 | 3 | 136 | 4 | 1 | 59 | 2 | 18 |
|  | Food/Plants/Supplements | 0 | 0 | 0 | 0 | 0 | 0 | 0 | 2 | 0 | 0 | 0 | 0 |
|  | Lifestyle | 0 | 0 | 0 | 0 | 0 | 1 | 0 | 0 | 1 | 0 | 0 | 0 |
|  | Procedure | 0 | 0 | 19 | 7 | 14 | 7 | 3 | 2 | 3 | 8 | 1 | 3 |
|  | Surgical | 0 | 0 | 11 | 3 | 1 | 0 | 0 | 0 | 0 | 6 | 22 | 0 |
|  | Wellness | 1 | 0 | 1 | 4 | 0 | 3 | 1 | 0 | 1 | 0 | 0 | 0 |
| BART Labelling strategy 7 | Behavioural | 4 | 0 | 3 | 0 | 4 | 0 | 0 | 0 | 0 | 0 | 0 | 0 |
|  | Biological Treatment | 0 | 11 | 4 | 1 | 118 | 2 | 0 | 17 | 1 | 17 | 0 | 0 |
|  | Delivery of Health Care Services | 0 | 0 | 14 | 0 | 0 | 2 | 0 | 0 | 3 | 0 | 2 | 0 |
|  | Device | 0 | 0 | 1 | 0 | 2 | 0 | 0 | 0 | 0 | 1 | 1 | 0 |
|  | Drugs | 0 | 4 | 4 | 1 | 271 | 0 | 0 | 10 | 0 | 34 | 0 | 0 |
|  | Education | 2 | 0 | 1 | 0 | 1 | 5 | 0 | 0 | 0 | 0 | 0 | 0 |
|  | Exercise Therapy | 6 | 1 | 20 | 28 | 13 | 6 | 133 | 8 | 0 | 62 | 3 | 17 |
|  | Food/Plants/Supplements | 0 | 0 | 0 | 0 | 0 | 0 | 0 | 0 | 0 | 0 | 0 | 0 |
|  | Lifestyle | 0 | 0 | 1 | 0 | 0 | 0 | 0 | 0 | 1 | 0 | 0 | 0 |
|  | Procedure | 6 | 0 | 36 | 19 | 40 | 14 | 13 | 9 | 8 | 27 | 11 | 5 |
|  | Surgical | 0 | 0 | 2 | 1 | 1 | 1 | 1 | 1 | 0 | 3 | 9 | 0 |
|  | Wellness | 1 | 0 | 2 | 0 | 2 | 1 | 0 | 0 | 0 | 0 | 1 | 0 |
